# Serum Activity of Platelet-Activating Factor Acetylhydrolase Is a Potential Clinical Marker for Leptospirosis Pulmonary Hemorrhage

**DOI:** 10.1371/journal.pone.0004181

**Published:** 2009-01-15

**Authors:** Junwei Yang, Yixuan Zhang, Jing Xu, Yan Geng, Xiaoying Chen, Hongliang Yang, Shengnian Wang, Hengan Wang, Xucheng Jiang, Xiaokui Guo, Guoping Zhao

**Affiliations:** 1 Laboratory of Synthetic Biology, Institute of Plant Physiology and Ecology, Shanghai Institutes for Biological Sciences, Chinese Academy of Sciences, Shanghai, China; 2 School of Life Science and Biopharmaceutical, Shenyang Pharmaceutical University, Shenyang, China; 3 Department of Medical Microbiology and Parasitology, Department of Pathology and Department of Animal Biotechnology, Shanghai Jiao Tong University, Shanghai, China; 4 Shanghai Institute of Health Sciences, Shanghai, China; 5 National Engineering Center for Biochip at Shanghai, Shanghai, China; 6 Shanghai-MOST Key Laboratory for Health and Disease Genomics, Chinese National Human Genome Center at Shanghai, Shanghai, China; 7 Department of Microbiology and Li Ka Shing Institute of Health Sciences, The Chinese University of Hong Kong, Prince of Wales Hospital, Shatin, New Territories, Hong Kong SAR, China; University of Hyderabad, India

## Abstract

Pulmonary hemorrhage has been recognized as a major, often lethal, manifestation of severe leptospirosis albeit the pathogenesis remains unclear. The *Leptospira interrogans* virulent serogroup Icterohaemorrhagiae serovar Lai encodes a protein (LA2144), which exhibited the platelet-activating factor acetylhydrolase (PAF-AH) activity *in vitro* similar to that of human serum with respect to its substrate affinity and specificity and thus designated L-PAF-AH. On the other hand, the primary amino acid sequence of L-PAF-AH is homologous to the α1-subunit of the bovine brain PAF-AH isoform I. The L-PAF-AH was proven to be an intracellular protein, which was encoded unanimously and expressed similarly in either pathogenic or saprophytic leptospires. Mongolian gerbil is an appropriate experimental model to study the PAF-AH level in serum with its basal activity level comparable to that of human while elevated directly associated with the course of pulmonary hemorrhage during severe leptospirosis. Mortality occurred around the peak of pulmonary hemorrhage, along with the transition of the PAF-AH activity level in serum, from the increasing phase to the final decreasing phase. Limited clinical data indicated that the serum activity of PAF-AH was likely to be elevated in the patients infected by *L. interrogans* serogroup Icterohaemorrhagiae, but not in those infected by other less severe serogroups. Although L-PAF-AH might be released into the micro-environment *via* cell lysis, its PAF-AH activity apparently contributed little to this elevation. Therefore, the change of PAF-AH in serum not only may be influential for pulmonary hemorrhage, but also seems suitable for disease monitoring to ensure prompt clinical treatment, which is critical for reducing the mortality of severe leptospirosis.

## Introduction

Leptospirosis continues to be a leading zoonotic infection throughout the world [1]. Pathogenic leptospires infection caused a diverse array of clinical manifestations ranging from subclinical infection to undifferentiated febrile illness to jaundice, renal failure, and potentially lethal pulmonary hemorrhage [2], [3]. Pulmonary hemorrhage is the most frequent cause of death of leptospirosis [4]–[6].

So far, lipopolysaccharide (LPS) [7], [8], glycolipoprotein [9], [10], peptidoglycan [11], hemolysin [12], and other virulent factors have been considered contributing to the leptospira's pathogenicity. However, little is known about the substances and mechanisms responsible for the hemorrhage and hemoptysis involved in their pathogenicity. It is generally believed that neither thrombocytopenia nor diminution of hepatically synthesized clotting factors seen in human leptospirosis were sufficient to account for the bleeding diathesis observed *per se* [1]–[3]. In 2004, Nally *et al.* reported that an autoimmune process may be involved in the etiology of fatal pulmonary hemorrhage in leptospirosis [13].

Based on the annotation of *Leptospira interrogans*' genomic sequence (serovar Lai strain 56601) [14], we recognized that the open reading frame (ORF) of *la2144* in the bacterial chromosome I seems encoding a putative platelet-activating factor acetylhydrolase (PAF-AH). Its primary amino acid sequence exhibited significant levels of similarity to the α1-subunit of bovine brain PAF-AH isoform I (EC 3.1.1.47), a 29-kDa protein isolated from bovine brain soluble fraction [15]–[17]. PAF-AH isoform I is an intracellular heterotrimeric enzyme composed of three subunits with molecular mass of 29- (α1), 30- (α2), and 45-kDa (β) respectively. Both α1 and α2 are catalytic subunits, but the α1 alone is sufficient to catalyze the removal of the acetyl moiety at the *sn*-2 position of PAF to generate the biologically inactive lyso-PAF [17]. Thus, LA2144 might hydrolyse PAF through its PAF-AH activity, and it was speculated to be associated with the pulmonary hemorrhage during severe leptospirosis.

Here, the identification and biochemical characterization of this leptospiral PAF-AH is firstly reported. By experimental infection to a Mongolian gerbil model, we found that although leptospiral PAF-AH contributed little to the change of PAF-AH level in gerbil sera during severe leptospirosis, the PAF-AH levels in serum were elevated along with the development of clinical manifestation, particularly, the whole course of pulmonary hemorrhage.

## Results

### The LA2144 is homologous to the PAF-AH isoform I α1-subunit

**Figure 1:**
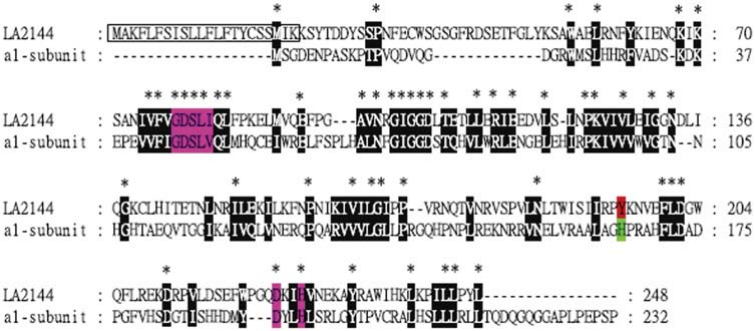
Alignment of the amino acid sequences of LA2144 and bovine PAF-AH isoform I α1-subunit. The alignment was generated by using ClustalW. Identical and similar amino acids were shaded. Identical amino acids were indicated by asterisks. The conserved motif Gly-Asp-Ser-Leu-Val/Ile, and the enzyme activity sites, Asp and His, were indicated in purple. Hypothetical signal peptide was boxed. Tyr^195^ of LA2144 was marked in red, and its relative amino acid at bovine PAF-AH isoform I α1-subunit was indicated in blue.

Deduced amino acid sequence of ORF LA2144 (amino acid residues 23–248) of *L. interrogans* serovar Lai was highly homologous (23% identity and 42% similarity) to that of the α1-subunit of PAF-AH isoform I (Figure 1). The α1 subunit of PAF-AH isoform I contains an essential serine residue within a motif which was perfectly conserved in LA2144 (Gly^78^-Asp^79^-**Ser^80^**-Leu^81^-Ile^82^). Besides, other two essential His^224^ and Asp^227^ residues were also conserved in LA2144, which may form a catalytic triad with Ser^80^ as in α1 (Figure 1). It thus suggested that LA2144 might have an “α1-subunit-like” function despite its unique N-terminal 22 residues (Figure 1) and the gene encoding LA2144 was thus tentatively designated *l-paf-ah* for leptospiral platelet-activating factor acetylhydrolase [14].

### The heterogeneously expressed L-PAF-AH demonstrated PAF-AH activity similar to that in the human serum

The *l-paf-ah* gene, with its original 66 nucleotides deleted from 5′ end, was cloned with an N-terminal His-tag fusion and expressed in *Escherichia coli* and then purified to homogeneity indicated by SDS-PAGE (Figure S1). The PAF-AH activity of the recombinant His-tag-L-PAF-AH protein (rL-PAF-AH) was assayed by employing the PAF artificial substrate [1-myristoyl-2-(4-nitrophenyl succinyl) phosphatidylcholine]. The kinetic parameters of the purified rL-PAF-AH was determined (Table 1) with its specific activity being 13.91 IU/mg (*kcat* = 6.61 s^−1^). Meanwhile, the *Km* of the human serum PAF-AH towards the artificial substrate was found to be 0.27 mM assayed by the same methodology (Table 1), which is in the same order of magnitude with that of the rL-PAF-AH (0.61 mM).

**Table 1:** Kinetic parameters of rL-PAF-AH, human serum and the specific activity of Y195H rL-PAF-AH.

Enzyme	*Km* (mM)	*kcat* (s^−1^)	*kcat/Km* (mM^−1^ s^−1^)	Specific activity (IU/mg)
**rL-PAF-AH**	**0.61**	**6.61**	**10.84**	**13.91**
**Human serum**	**0.27**			
**Y195H rL-PAF-AH**				**12.79**

All values represented means of results from duplicate experiments.

### L-PAF-AH is a cytosolic protein

Since the N-terminal (residues 1–22) of L-PAF-AH was predicted as a signal peptide (Figure 1), we tried to examine whether the L-PAF-AH was located on the surface of the cell. We failed to detect any PAF-AH activities by examining the resting live cells of *L. interrogans* serovar Lai strain 56601 although it was readily detected in the crude cell extracts (unpublished data). Therefore, the L-PAF-AH is unlikely an enzyme located outside of the cell.

**Figure 2:**
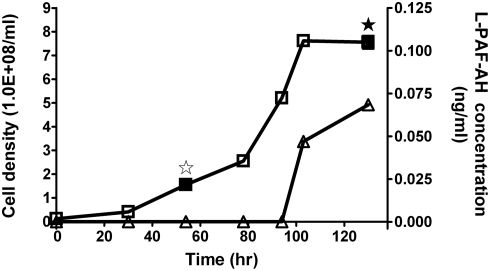
As an intracellular protein, L-PAF-AH was released into the culture medium *via* autolysis when entering the stationary phase. *L. interrogans* was grown in EMJH medium. Culture samples were obtained at 0, 30, 54, 78, 94, 103 and 130 hr, after initial inoculation. The cell densities (squares) were counted in a Petroff-Hausser counting chamber (Fisher) while the L-PAF-AH protein concentrations (triangles) in the culture supernatants were determined by ELISA. The standard deviations were in all cases below 10%. L-PAF-AH protein was further detected in selected cells crude extracts and culture supernatants (★, ⋆, ▪) by mass spectrometry. ★, identified in culture supernatants; ⋆, not identified in culture supernatants; ▪, identified in cells crude extracts.

We also tried to examine whether the L-PAF-AH in *L. interrogans* serovar Lai strain 56601 was secreted into the medium. Leptospiral culture of strain 56601 in EMJH medium was initiated with 10% inoculation of a mid-log-phase pre-culture and incubated under 28°C with gentle shaking. Aliquot samples of the culture were collected at various time points (0, 30, 54, 78, 94, 103, and 130 hr) over the incubation period up to the stationary phase (Figure 2). The culture samples collected at each time point were concentrated by lyophilization and examined by ELISA with anti-L-PAF-AH specific antibodies. The assay indicated that L-PAF-AH in the media was not detected until entering the early-stationary phase (103 hr) when bacterial cell lysis was observed (Figure 2). The concentration of L-PAF-AH in the media increased in the later stationary phase as more cells were lysed (130 hr) (Figure 2).

Employing the anti-L-PAF-AH specific antibodies, the L-PAF-AH protein was immunoprecipitated from both the cell crude extract of *L. interrogans* serovar Lai strain 56601 cultured in EMJH media and the culture supernatant from the same sample and then examined by mass spectrometry. L-PAF-AH was identified in the cytosolic fractions from samples collected at both the 54^th^ hr (log phase) and the 130^th^ hr (stationary phase) (Figure 2). However, it was only detected in the medium supernatant of the 130^th^ hr sample, but not in that of the 54^th^ hr sample. These results clearly demonstrated that L-PAF-AH is an intracellular protein, which is not released into the culture medium until the bacterial cells were autolysed.

### The *l-paf-ah* gene is present unanimously and expressed similarly in either pathogenic or saprophytic leptospires

Nine standard strains of different serovars commonly found in China and the avirulent strain of *L. interrogans* serovar Lai (Table 2) were selected to analyze their *l-paf-ah* gene including their probable promoter regions *via* PCR amplification followed by sequencing confirmation. The results indicated that the *l-paf-ah* gene is unanimously present in these strains with limited genetic variations. There was only one asynonymou single nucleotide variation (SNV) causing the Y195H amino acid switch (Table 2) found in the *l-paf-ah* gene out of maximal 8 SNVs from four leptospires (Figure S2). The recombinant Y195H L-PAF-AH (Y195H rL-PAF-AH) was able to hydrolyze PAF, and its specific activity was 12.79 IU/mg, similar to that of the L-PAF-AH from strain 56601 (Table 1).

**Table 2:** The amino acid^195^, RT-PCR and specific activity of PAF-AH in ten leptospires cultured in EMJH and Korthof media.

Strain	Amino acid^195^	RT-PCR a	RT-PCR b	Specific activity c (IU/10^10^ leptospira)	Specific activity d (IU/10^10^ leptospira)
*L. interrogans* serovar Lai strain 56601	Tyr	**+**	**+**	0.22±0.023	0.4±0.045
Avirulent variant strain of *L. interrogans* serovar lai	Tyr	**+**	**+**	0.18±0.015	0.38±0.05
*L. interrogans* serovar Javanica strain M 10	Tyr	**+**	**+**	0.20±0.021	0.48±0.06
*L. interrogans* serovar Canicola strain Lin	His	**+**	**+**	0.15±0.018	0.45±0.06
*L. interrogans* serovar Pomona strain Luo	His	**+**	**+**	0.14±0.017	0.35±0.03
*L. interrogans* serovar Linhai strain Lin 6	Tyr	**+**	**+**	0.17±0.023	0.40±0.05
*L. interrogans* serovar Hebdomadis strain P 7	His	**+**	**+**	0.25±0.03	0.37±0.03
*L. interrogans* serovar Paidjan strain L 37	Tyr	**+**	**+**	0.24±0.03	0.34±0.03
*L. biflexa* serovar Montevalerio	Tyr	**+**	**+**	0.28±0.025	0.48±0.05
*L. biflexa* serovar Anhui strain Zong 7	His	**+**	**+**	0.21±0.016	0.41±0.06

a RT-PCR detection of *l-paf-ah* mRNA from leptospires cultured in EMJH medium. +, positive result.

b RT-PCR detection of *l-paf-ah* mRNA from leptospires cultured in Korthof medium. +, positive result.

c Specific activity of leptospires cultured in EMJH medium.

d Specific activity of leptospires cultured in Korthof medium.

For expression patterns, the *l-paf-ah* mRNAs were detected in all of the ten leptospira strains grown in EMJH and Korthof media *via* RT-PCR (Table 2). We also determined the specific activity in the cell crude extracts. As shown in Table 2, the specific activities were similar among ten pathogenic and saprophytic leptospires cultured in EMJH or Korthof media. These results demonstrated that L-PAF-AHs are conserved in both their primary structure and the expression patterns in eight pathogenic leptospires and the two saprophytic leptospires.

### Mongolian gerbil as the experimental model for analyzing serum PAF-AH related symptoms

**Figure 3:**
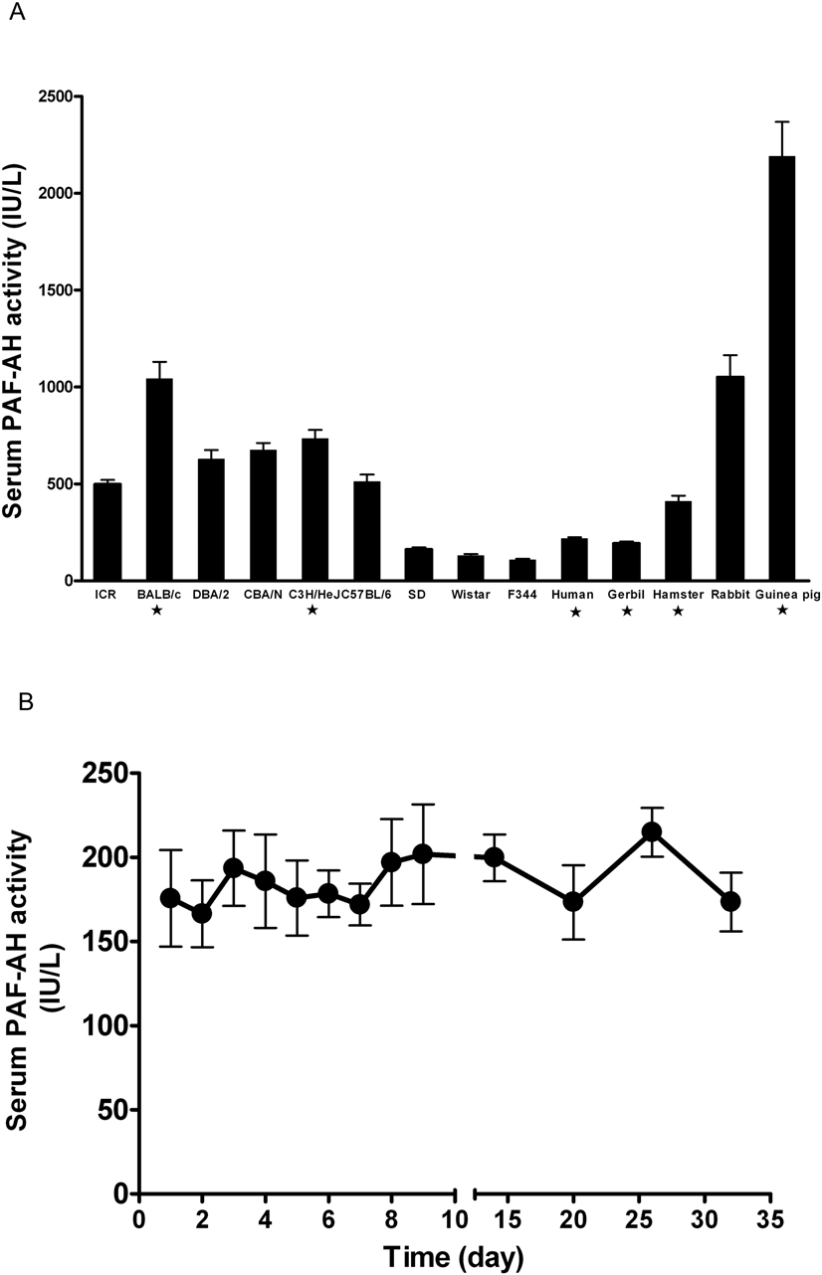
Serum PAF-AH levels of healthy human individuals and animal species (A), and the serum PAF-AH levels of normal gerbils (n = 6) on the serial 32 days (B). Bars indicated the standard error. ★, human or animal species commonly used for experimental study of leptospirosis.

Among the various animal species used for experimental studies of leptospirosis, Mongolian gerbil [18], [19] has been known for its lethal infection to leptospira of many serovars with symptoms of pulmonary hemorrhage. We determined the PAF-AH levels in the sera of healthy mice (ICR, BALB/c, CBA/N, C57BL/6, DBA/2, and C3H/HeJ), rats (Fischer 344, Sprague-Dawley, and Wistar), golden hamster, guinea pig, Mongolian gerbil, and New Zealand rabbit (Figure 3A). Serial daily measurements over 32 days indicated that the enzyme level was stable in healthy gerbils (Figure 3B), similar to that of human beings (Figure 3A). Therefore, Mongolian gerbil was selected as the experimental model.

### Mortality of the *L. interrogans* infected gerbil was strongly associated with pulmonary hemorrhage

**Figure 4:**
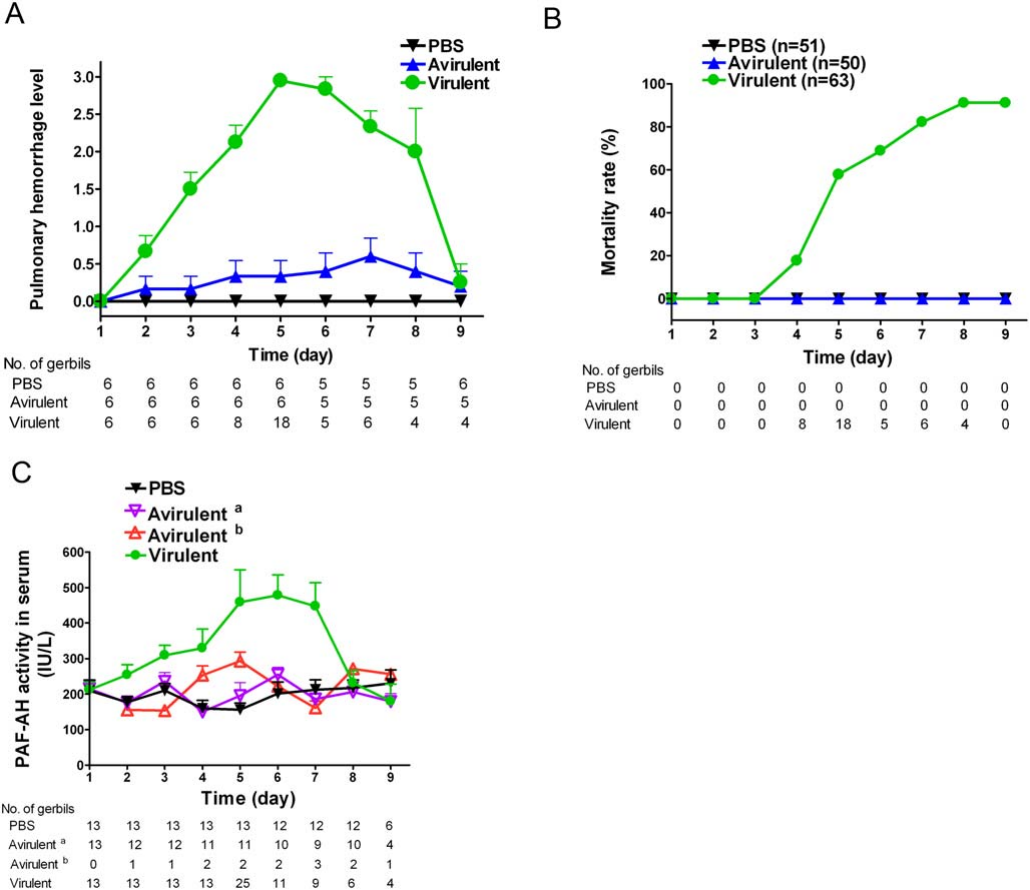
Time course of pulmonary hemorrhage (A), mortality rate (B) and PAF-AH level in serum (C) of gerbils inoculated with PBS, the avirulent strain of *L. interrogans* serovar Lai and *L. interrogans* serovar Lai. PBS, injected with PBS alone; avirulent, injected with the avirulent strain of *L. interrogans* serovar Lai; virulent, injected with *L. interrogans* serovar Lai. (A) The measurement and plot of pulmonary hemorrhage referred to *Materials and Methods*. (C) Avirulent ^a^, no pulmonary hemorrhage observed in the gerbils injected with the avirulent strain of *L. interrogans* serovar Lai; avirulent ^b^, pulmonary hemorrhage observed in the gerbils injected with the avirulent strain of *L. interrogans* serovar Lai. At indicated time, number of animals was shown under the figures. Bar indicated the standard error.

The leptospirosis in gerbil model was designed to study the association of PAF-AH level in serum and pulmonary hemorrhage during the whole course of *L. interrogans* serovar Lai infection (Figure S3) with an avirulent strain (Figure S4) as the negative control. Gerbils intraperitoneally injected with the virulent strain began to show loss of mobility, and general unkemptness from day 2 to day 3, and on the 9^th^ day, the activities of all survived animals were back to normal. Animals in the virulent group began to show pulmonary hemorrhage on day 2 after inoculation, and pulmonary hemorrhage became much more severe on the 5^th^ day. Then, pulmonary hemorrhage became less and less severe and totally returned to normal on the 9^th^ day (Figure 4A). Dying of the model animals began to occur on day 4, and the peak of mortality was observed on the 5^th^ day (Figure 4B). Autopsy of all animals that died on day 5 and 6 were shown to bear jaundice and pulmonary hemorrhage. For animals died on the 4^th^, 7^th^ and 8^th^ day, pulmonary hemorrhage was the only typical pathological symptom observed (Figure 4B).

Autopsy of animals sampled from day 1 to day 9 of the PBS group demonstrated no hemorrhage (Figure 4B). However, minor pulmonary hemorrhage (Figure 4B) without mortality (Figure 4A) was observed in gerbils infected by the avirulent strain from day 2 to day 9, or in gerbils infected by the virulent strain sampled on day 2 and day 3. These results implicated that severe pulmonary hemorrhage was strongly associated with the death of infected models, in other words, all gerbils died with pulmonary hemorrhage. On the other hand, more gerbils died during the increasing phase of pulmonary hemorrhage (n = 26, sum for day 4 and day 5) than the decreasing phase (n = 15, sum for day 6 to day 8), and most gerbils (n = 18) died on the 5^th^ day when the pulmonary hemorrhage was the most severe (Figure 4A and 4B).

### The change of PAF-AH level in serum was directly associated with the pulmonary hemorrhage

PAF-AH level in serum of the animals injected with the virulent strain began to increase from day 2, reached the peak on day 5, and then decreased to normal on the 8^th^ day and these elevations were statistically significant (*P*<0.01) (Figure 4C). In contrast, there was no significant difference for the PAF-AH level in sera of groups injected with either the PBS or the avirulent strain (Figure 4C). The only exception was that on the 4^th^ and 5^th^ day, the PAF-AH level in serum of the avirulent strain group with pulmonary hemorrhage was slightly higher than that of the PBS group and the avirulent group without pulmonary hemorrhage. Therefore, PAF-AH level in serum is directly associated with the pulmonary hemorrhage level along the course of disease.

**Figure 5:**
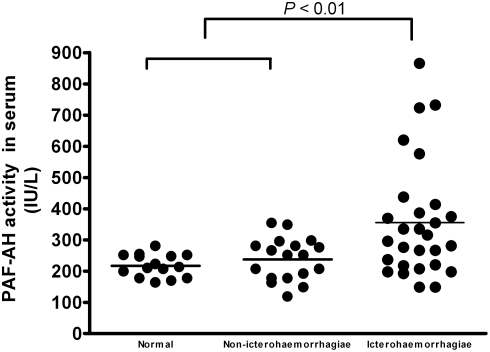
PAF-AH levels in serum of patients with leptospirosis and healthy adult individuals. Normal (n = 15), healthy human group; non-icterohaemorrhagiae (n = 18), leptospirosis patients infected by *L. interrogans* serogroup Pyrogenes (n = 3), Autumnalis (n = 3), Pomona (n = 3), Hebdomadis (n = 4) and Canicola (n = 5), other than *L. interrogans* serogroup Icterohaemorrhagiae; icterohaemorrhagiae (n = 28), leptospirosis patients infected by *L. interrogans* serogroup Icterohaemorrhagiae. Horizontal bars represented the mean value for each group.

PAF-AH assay was also carried out employing serum samples from some leptospirosis patients. The data shown in Figure 5 indicated that the serum activity of PAF-AH was elevated in the patients infected by *L. interrogans* serogroup Icterohaemorrhagiae, but not in those infected by *L. interrogans* serogroup Pyrogenes, Autumnalis, Pomona, Hebdomadis or Canicola. Although more clinical data are required for proof, it suggested that the change of PAF-AH activity in patients' serum might be associated with different serogroups of the infectious leptospires, which may or may not lead to the life-threatening pulmonary hemorrhage.

### L-PAF-AH contributed little PAF-AH activity to the increase of PAF-AH level in serum in the severe leptospirosis

**Figure 6:**
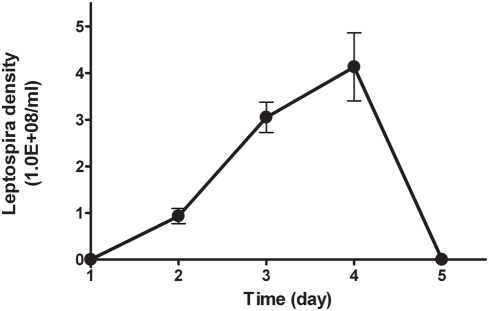
Blood leptospira level of eight gerbils infected by *L. interrogans* serovar Lai. 0 represented that leptospira was not observed. Bar indicated the standard error.

Although L-PAF-AH was identified as an intracellular protein, it could be released into the blood circulation after leptospira cells were lysed, and then led to the elevation of PAF-AH level in serum. We tried to make a rough estimation of L-PAF-AH released to the serum by measuring its level in an *in vitro* serum culture (Korthof medium). The specific activity of L-PAF-AH from *L. interrogans* serovar Lai grown in Korthof medium was approximately 0.4 IU/10^12^ leptospires (Table 2). The average level of PAF-AH in serum from gerbil infected with the virulent strain was 418.3 IU/L. Therefore, comparing to the 203.7 IU/L level of the PBS group, the average elevation after infection was 214.6 IU/L. For gerbils with 40 to 70 g of body weight, there was approximately 5 ml total circling blood [20]. Therefore, if the L-PAF-AH was the only factor for the elevation of PAF-AH level in serum, the leptospira density would be 1.1×10^12^/ml (5.4×10^12^ bacteria in 5 ml gerbil circulating blood). However, the highest leptospira density measured in the gerbil circulating blood was approximately 4×10^8^/ml during the course of disease (Figure 6).

To exclude the possibility of higher level of L-PAF-AH expression from *L. interrogans* in gerbils than that cultured in Korthof medium, anti-L-PAF-AH rabbit polyclonal serum and murine monoclonal antibodies were used to detect L-PAF-AH in the sera of gerbils with pulmonary hemorrhage employing both ELISA and immunoprecipation followed by mass spectrometry. There was no detectable L-PAF-AH in the 500 µl sera collected either during the increasing phase for PAF-AH in serum (on the 2^nd^, 3^rd^, 4^th^, 5^th^ and 6^th^ days) or during the decreasing phase (on the 7^th^, 8^th^ and 9^th^ days) (Figure S5). Therefore, L-PAF-AH might contribute little to the increase of PAF-AH level in serum for the severe leptospirosis.

## Discussion

When L-PAF-AH was firstly recognized by its deduced amino acid sequence [14] and simulated tertiary structure (unpublished data) similarity to the α1-subunit of the mammalian PAF-AH isoform I *via* the annotation of *L. interrogans* genome, its enzymatic activity was identified employing an artificial substrate used for assaying the serum enzyme [21]. Although, so far, there is no complete data available for enzyme kinetic parameters of the serum PAF-AH towards this artificial substrate, its Km was determined in this study, which is similar to that of the L-PAF-AH. Therefore, in contrast to the analogous activity found in a bifunctional CMP-*N*-acetylneuraminic acid synthetase (CS) encoded by a pathogenic *E. coli* K1 as one of its two catalytic domains [22], this L-PAF-AH is an independent single-chain enzyme, with its structure similar to that of the mammalian isoform I α1-subunit while its substrate specificity being different from that of the *E. coli* CS but similar to the serum counterpart enzyme.

At least three types of PAF-AH have been identified in mammals, *i.e.*, the intracellular types I and II, and a serum (plasma) type. The PAF-AH II has its substrate specificity similar to the serum PAF-AH. Both enzymes can hydrolyze phospholipids with short to medium length *sn-2* acyl chains including truncated ones derived from oxidative cleavage of long chain polyunsaturated fatty acyl groups [23], such as 1-myristoyl-2-(4-nitrophenylsuccinyl)-phosphatidylcholine [21]. On the other hand, PAF-AH isoform I hydrolyzes the *sn-2* ester bond in PAF-like phospholipids with a marked preference for very short acyl chains, typically acetyl. The α1-subunit of PAF-AH isoform I has a strong preference for acetyl groups attached to the glycerol backbone of PAF [24], and this specificity is ascertained to the Leu^48^, Leu^194^ and Thr^103^ residues of the enzyme by forming contact with the methyl group of acetate [17]. The PAF-AH domain of the *E. coli* CS is highly similar to the α1-subunit of PAF-AH isoform I with respect to both its amino acid sequence and the computer simulated 3-D structure. Thus, it is no surprise that the PAF-AH domain of the *E. coli* CS exhibited similar substrate specificity as that of the α1-subunit [22]. The amino acid residues corresponding to Leu^48^, Leu^194^ and Thr^103^ of the α1-subunit were also conserved in the *E. coli* CS (identified as Leu^258^, Leu^399^ and Thr^308^ based on three-dimensional modeling) [22].

In contrast, our results showed that L-PAF-AH, which is again, highly similar to the α1-subunit of PAF-AH isoform I with respect to the primary amino acid sequences (Figure 1) and the simulated 3-D structure (unpublished data), could hydrolyze the 1-myristoyl-2-(4-nitrophenylsuccinyl)-phosphatidylcholine (Table 1). Through alignment of the primary amino acid sequences of L-PAF-AH with the bovine PAF-AH isoform I α1-subunit, the putative corresponding amino acid residues involved in this substrate specificity was deduced as Leu^81^, Ile^226^ and Gly^132^ in correspondence to the Leu^48^, Leu^194^ and Thr^103^ of the isoform I α1-subunit (Figure 1), which was different from that either in the PAF-AH domain of the *E. coli* CS or in the α1-subunit of PAF-AH isoform I. In conclusion, the L-PAF-AH is a unifunctional PAF-AH enzyme encoded by bacteria with its substrate specificity different from that of the above mentioned *E. coli* PAF-AH domain or α1-subunit of PAF-AH isoform I, probably due to the change of the conserved substrate binding related amino acid residues.

We also proved that L-PAF-AH was an intracellular enzyme conserved among all species of *Leptospira*, pathogenic or saprophytic. The *l-paf-ah* gene is unanimously present in ten pathogenic and saprophytic leptospires, and they were primarily expressed at similar levels while culturing in EMJH or Korthof media detected by RT-PCR (Table 2). The specific activities of these cultures were also similar among pathogenic and saprophytic leptospires grew under the same conditions (Table 2). These results implicated that L-PAF-AH is involved in the physiological activities of leptospires.

Employing L-PAF-AH specific monoclonal and polyclonal antibodies in combination, we were able to access the contribution of L-PAF-AH in the total PAF-AH level in serum during hemorrhage stage of severe leptospirosis in gerbil experimental model. Although the PAF-AH activity in serum was elevated in the experimental leptospirosis, the contribution of L-PAF-AH was very low (estimated, 0.07%). On the other hand, it is still possible that the L-PAF-AH released from autolysed leptospires during mid- and late-phase of infection may function as a virulent factor within the local niche of small blood capillaries of the alveoli [14] as its substrate, PAF, a potent lipid messenger, participates in a variety of physiological events including platelet aggregation to maintain the blood homeostasis [25].

Recent reports suggested that serum PAF-AH might play an anti-inflammatory role in human diseases, such as atherosclerosis [26], asthma [27], and septic shock [28], by preventing the accumulation of PAF and PAF-like oxidized phospholipids. However, the simulation the role of serum PAF-AH in human diseases in different animal-models provided controversial outcomes even for the same disease. For instance, it was reported that LPS increases serum PAF-AH activity in Syrian hamsters [29]. However, in the Swiss and C57BL6 mouse models of endotoxemia administrated by LPS, serum activity of PAF-AH is significantly decreased within 24 hr after the challenge and then returns to baseline in surviving animals [30]. Thus, the nature of the animal selected for *in vivo* investigation was found to markedly affect the results. It would, therefore, be of interest to explore the suitable animal to simulate the role of human serum PAF-AH.

The present study first investigated the serum PAF-AH levels of the commonly used experimental rodent and rabbit species, particularly guinea pig [13], [31], [32] and golden hamster [33], the most common models for the leptospirosis studies and found that only Mongolian gerbil has its normal serum PAF-AH level similar to that of human (Figure 3A). Furthermore, the patterns of the change of PAF-AH level in serum during the course of severe leptospirosis were similar in gerbils and patients (Figure 5 and Figure S6), including the levels of elevation. Therefore, gerbil seems to be the best model animal among the rodent and rabbit species to reflect the role of serum PAF-AH in the human diseases.

Platelet aggregation as well as platelet adhesion and subsequent plug formation at the site of injury plays a major role in the control of vascular hemostasis. Decreases in PAF levels *in vivo*, leading to decreased ability of platelet aggregation, can profoundly influence vascular hemostasis [25], [34]. The acetyl group at the sn-2 position of its glycerol backbone is essential for its biological activity, and its deacetylation product, lyso-PAF, loses biological activity. Comparing PAF-AH to the control, its changes in serum were closely associated with the whole course of pulmonary hemorrhage (Figure 4B and 4C). In human, the PAF-AH levels in serum of leptospirosis patients infected by *L. interrogans* of serogroup Icterohaemorrhagiae were found significantly higher than that of either normal control or patients infected by non-icterohaemorrhagiae leptospires (*P*<0.01) (Figure 5). These findings could be directly related to the possible decrease of the blood platelet activation activity of PAF caused by the elevated PAF-AH catalyzed hydrolysis. Eventually, pulmonary hemorrhage happens, of course, including the effects of the other virulent factors, such as LPS. Thus, the PAF-AH in serum might play an important role in the pulmonary symptom in severe leptospirosis although its mechanism is yet to be learnt along with the confirmation of this hypothesized function.

Practically, PAF-AH level in serum seems more likely a suitable biochemical marker for monitoring the occurrence of the lethal pulmonary hemorrhage in cases of severe leptospirosis. Most human fatalities in leptospirosis are due to hemorrhage, and pulmonary involvement is the main type of hemorrhage in the host [1]. Clinical course of pulmonary hemorrhage has been known to be developed so quickly that doctors did not have enough time to provide the suitable therapy, while early treatment could reduce most of the death for severe leptospirosis patients [1]–[3]. Thus, the need for developing some convenient monitoring strategies has become even more critical now. The significant direct association of the PAF-AH activities in serum with the pulmonary hemorrhage implied that it would be one biochemical marker for monitoring the level of risk for pulmonary hemorrhage development along the course of the disease, which in turn, may direct the doctors to apply different therapeutic strategies for individual patients suffered in different disease phases to reduce the mortality rate of the severe leptospirosis. In our opinion, suitable antibiotics application for different duration of the disease with different PAF-AH level in serum might be suggested to support the present existed standards, such as doxycycline for both prophylaxis and mild disease [35], [36], ampicillin and amoxicillin for mild disease, whereas penicillin G and ampicillin for severe disease [37]. Besides, because PAF-AH is also considered a regulatory molecule for immune responses, appropriate application of some immune modulation drugs at certain duration of the severe leptospirosis might be considered, at least for animal model analysis. Finally, some assistant therapies, such as hospital admission and close observation, should be required for the severe patients at their early stage of the disease course. Thus, our observations have provided a potential clinical molecular marker for prospective clinical evaluation, by which, the manifestation course leading to pulmonary hemorrhage might be monitored to reduce the mortality of the severe leptospirosis.

To our knowledge, it is the first time that increased PAF-AH level in serum was reported for sepsis. Graham *et al.* reported decreased PAF-AH serum activity in blood samples from septic patients and the half-life of PAF was prolonged in the serum of dying septic patients compared to survivors or normal volunteers [38]. Furthermore, in mouse models, serum activity of PAF-AH is significantly decreased within 24 hr after the challenge and then returns to baseline in surviving animals [30]. As leptospirosis shows typical sepsis symptoms including chills, headache, myalgia, abdominal pain, conjunctival suffusion, and less often a skin rash [39], it was surprising to observe the elevation of PAF-AH in serum in severe leptospirosis patients (Figure 5) and gerbil models (Figure 4C). In addition, the PAF-AH levels in serum of dying gerbils, most of which showed the severest pulmonary hemorrhage, were significant higher than the mean level of total gerbils infected the virulent strain (Figure S6). Considering that leptospires are different from either Gram-positive or Gram-negative bacteria with basically unknown mechanisms of pathogenicity or virulence [1], the modulation of PAF-AH in serum might be different between the leptospirosis patients *vs.* other sepsis patients, for most of them were infected by typical Gram-positive or Gram-negative pathogenic bacteria [30], [38]. Thus, this changing pattern of PAF-AH in serum, so-far unique for severe leptospirosis may distinguish it from other reported sepses [30], [38], which may eventually help us to further understand the pathogenesis of leptospirosis. Moreover, as different sepses may have different response of PAF-AH activity, the exact role of serum PAF-AH in different kinds of sepses must be answered before undertaking any related medication, *i.e.* administration of recombinant serum PAF-AH [40], [41].

Intracellular isoforms of PAF-AH exist and are found in liver, kidney and brain [42]—tissues involved in leptospiral complications and multiple organ failure [1]. It is possible that not all of the activities measured in the blood of patients and gerbils with leptospirosis were from the secreted, extracellular form of PAF-AH—the resident enzyme of normal serum—but also included intracellular activity that leaked from injured or dying cells. Therefore, one of our next challenges would be to identify which isoform of the host PAF-AH actually makes the major contribution of elevation and explore the elevation mechanism.

## Materials and Methods

### Bacterial strains, media, and growth conditions

The avirulent strain of *L. interrogans* serovar Lai was kindly provided by Professor Isabelle Saint Girons (Unité de Bactériologie Moléculaire et Médicale, Institut Pasteur, France). *L. interrogans* serovar Lai strain 56601 and other leptospiral strains (Table 2) used in this study are maintained by the National Institute for Communicable Disease Control and Prevention, Chinese Center for Disease Control and Prevention, China. Leptospires were cultivated in liquid EMJH or Korthof [39] medium at 28°C under aerobic conditions, and the bacterial cell density of the culture was counted in a Petroff-Hausser counting chamber (Fisher). Only the mid-log phase cells were used for experiments.

*E. coli* strains DH5α and BL21 (DE3) pLysS (Novagen) were used for heterogeneous gene cloning and expression, respectively. They were routinely propagated at 37°C in LB medium [43]. Growth medium was supplemented with 100 µg/ml ampicillin or 50 µg/ml kanamycin when required.

### Bioinformatic analysis

Sequence similarity search was performed using the program BLAST. The multiple alignment of amino acid sequences was performed by ClustalW. The signal peptide was predicted by SignalP 3.0.

### Recombinant plasmid construction, protein expression and purification

*L. interrogans* gene *l-paf-ah* was synthesized by PCR with strain 56601 or serovar Canicola strain Lin genomic DNAs as the templates (Table 2). PCR primers were designed as following: forward, 5′-CATATGTTGATTAAGAAATCATATACG-3′; reverse, 5′-TTAAAGATAGGGTAAAAGTATCGGT-3′. The desired gene fragment was cloned into pET-28b, and confirmed by DNA sequencing. Protein expression was induced by 0.4 mM isopropyl-D-thiogalactopyranoside at 28°C for 4 hr when A600 values reached 0.6–0.8. Protein purification was applied to Ni-NTA column. Protein concentration was determined by the bicinchoninic acid method, with bovine serum albumin as the standard.

### Enzyme activity assay

Measurement of PAF-AH activity was performed as described [21] employing the Azwell Auto PAF-AH kit (Azwell): PAF-AH hydrolyzes the sn-2 position of the substrate [1-myristoyl-2-(4-nitrophenyl succinyl) phosphatidylcholine], a PAF (1-alkyl-2-acetyl phosphatidylcholine/1-*O*-alkyl-2-acetyl-*sn*-glycero-3-phospholine) analogue with a 4-nitrophenyl substituent, resulting in generating 4-nitrophenyl succinate, which is immediately degraded to 4-nitrophenol and subsequently measured spectrophotometrically at 405 nm. The activity is expressed in IU/L; 1 IU of PAF-AH hydrolyses 1 µmol of substrate in 1 min. The enzyme kinetics data were collected employing the purified rL-PAF-AH while human serum samples were used for the Km determination of human serum PAF-AH.

### Production of anti-L-PAF-AH rabbit polyclonal serum and murine monoclonal antibodies (MoAbs)

New Zealand rabbits were immunized with rL-PAF-AH. Equal amounts of adjuvant and antigen solution were mixed thoroughly for each inoculation. Subcutaneous injections of 200 µg of antigen per rabbit were carried out. First immunizations were done with complete Freund's adjuvant (Invitrogen) and subsequent immunizations were done employing incomplete Freund's adjuvant (Invitrogen) on days 14, 28 and 35. Rabbits were bled on the 1st or 2nd weeks after the last booster.

BALB/c mice were immunized intraperitoneally with purified rL-PAF-AH as previously reported [44]. The generated hybridoma cells were screened for antibody production by ELISA using microtiter plates coated with rL-PAF-AH. Positive hybridomas without reactivity against gerbil serum were selected, and injected into the pristane-primed BALB/c mice. After 3 weeks, ascitic fluid was collected and stored at −20°C. Murine MoAbs were purified from ascitic fluid on protein A-Sepharose 4B. The specificity of antibody-antigen interaction distinguishing the L-PAF-AH and the gerbil serum PAF-AH was further tested with ELISA.

### Antigen-capture ELISA

Antigen-capture ELISA was performed by following standard protocol described elsewhere in detail [45]. Anti-L-PAF-AH murine MoAb and rabbit polyclonal antibody were used as capture and detection antibodies for the L-PAF-AH, respectively.

### Immunoprecipitation and protein identification by mass spectrometry

Cell extract (1 ml), or culture supernatant (4 ml) of *L. interrogans* serovar Lai strain 56601, or serum (500 µl) were mixed with 5 µl of rabbit polyclonal serum rotating at 4°C for 2 hr, and incubated for 1 hr with 20 µl of protein A-Sepharose. Beads were washed (50 mM Tris, pH 7.5, 150 mM NaCl, 5 mM EDTA, 1% Triton X-100, 1 mM PMSF, 10 µg/ml Aprotinin and 10 µg/ml Leupeptin), and bound protein was eluted with Laemmli sample buffer and separated by SDS-PAGE. The approximate 20 kDa to 30 kDa band was incised, digested by trypsin, and identified by mass spectrometry shotgun analysis as described previously [46]. Nine peptides, some of which were overlapped, were identified through mass spectrometry shotgun analysis. Although the predicted signal peptide was not identified, the authenticity of the protein precipitated was confirmed.

### Animal study

Male mice (ICR, BALB/c, CBA/N, C57BL/6, DBA/2, and C3H/HeJ), rats (Fischer 344, Sprague-Dawley, and Wistar), golden hamster, guinea pig, Mongolian gerbil, and New Zealand rabbit, one to two months of age, were from Shanghai Laboratory Animal Center, China. Blood was collected by cardiac puncture, and serum PAF-AH level was detected to screen potential experimental animal.

Male Mongolian gerbils, two months of age, were used in the leptospira infection experiment. To exclude the effect of EMJH medium, spirochetes grown in EMJH medium were pelleted by centrifugation, resuspended in 0.01 M phosphate-buffered saline (PBS), repelleted, and washed twice in PBS. Gerbils were injected intraperitoneally with 1 ml PBS alone (negative control), PBS with the avirulent strain of *L. interrogans* serovar Lai (5×10^8^) and PBS with *L. interrogans* serovar Lai (5×10^8^). The animal study design was shown in Figure S3. Seven gerbils in each group were not euthanized until to the 9^th^ day after infection, and blood was collected from the tail. The other gerbils in each group were euthanized on the 1^st^, 2^nd^, 3^rd^, 4^th^, 5^th^, 6^th^, 7^th^, 8^th^ and 9^th^ day after infection, and blood was collected by cardiac puncture. All animal studies were approved by the Animal Research Committee of the Chinese National Human Genome Center at Shanghai.

### Measurement of pulmonary hemorrhage

The pulmonary hemorrhage was observed and measured as previously described [47] with slight modifications. The scores of 0, 1, 2 and 3 were defined to illustrate the level of severeness of the manifestation as absence of pulmonary hemorrhage, hemorrhagic focus (minimal), hemorrhagic area (moderate) and hemorrhagic diffusion (marked), respectively.

### Blood concentration of leptospires

Approximately 0.2 ml of blood was taken via the tail, and was left to stand in a heparinized Dreyer agglutination tube until the erythrocytes had settled sufficiently (10 min) to allow the leptospires in a drop of the supernatant to be counted in a Petroff-Hausser counting chamber (Fisher).

### Nucleotide sequence accession numbers

The nucleotide sequences of the *l-paf-ah*s and the corresponding upstream regions from nine leptospira strains were deposited in GenBank. The accession numbers are EF191070 for the avirulent strain of *L. interrogans* serovar Lai, EF191073 for *L. interrogans* serovar Javanica strain M 10, EF191069 for *L. interrogans* serovar Canicola strain Lin, EF191074 for *L. interrogans* serovar Pomona strain Luo, EF191071 for *L. interrogans* serovar Linhai strain Lin 6, EF191072 for *L. interrogans* serovar Hebdomadis strain P 7, EF191066 for *L. interrogans* serovar Paidjan strain L 37, EF191068 for *L. biflexa* serovar Montevalerio, and EF191067 for *L. biflexa* serovar Anhui strain Zong 7.

### RT-PCR

Total RNA from mid-log phase leptospires cultured in EMJH or Korthof medium (Table 2) was extracted by using Trizol Reagent (Invitrogen). RNase-free DNase-treated RNA was hybridized to random hexamer primers, and cDNA was synthesized with AMV reverse transcriptase as specified by the manufacturer (Promega). The cDNA was amplified with *Taq* DNA polymerase (Takara) with gene-specific primer pair. The primers were the same as the pair of primers used for recombinant *l-paf-ah* plasmid construction.

### Patients and control individuals

Serum samples were collected in 2008 from 46 patients with confirmed leptospirosis from Anhui Center for Disease Control and Prevention, China. Diagnosis was confirmed by the microagglutination test (MAT) with the following criteria: a four-fold or greater rise in titer between paired serum samples; seroconversion (initial MAT titer of <100 increasing to ≥200); or a single serum titer ≥400 were considered positive. Among the 46 leptospirosis patients, 28 patients were infected by *L. interrogans* serogroup Icterohaemorrhagiae. The other 18 patients were infected by *L. interrogans* serogroup Pyrogenes (n = 3), Autumnalis (n = 3), Pomona (n = 3), Hebdomadis (n = 4) and Canicola (n = 5). Control serum samples were collected from 15 healthy individuals. Written informed consent was obtained from each participant prior to the collection of blood according to protocols approved by the Chinese National Human Genome Center at Shanghai.

### Ethics statement

This human study was conducted according to the principles expressed in the Declaration of Helsinki. Written informed consent was obtained from each participant prior to the collection of blood according to protocols approved by the Chinese National Human Genome Center at Shanghai.

All animals were handled in strict accordance with good animal practice as defined by the relevant local animal welfare bodies, and all animal work was approved by the Animal Research Committee of the Chinese National Human Genome Center at Shanghai.

### Statistics

Data were analyzed with Graph-Pad Prism, version 2.0 (GraphPad Software). Data were presented as mean values±SEM. Statistical analyses were performed using one way analysis of variance (ANOVA).

## Supporting Information

Figure S1 Purification of His-Tag fusion protein L-PAF-AH in the E. coli BL21 (DE3) pLysS cells monitored with the 15% SDS-PAGE. Lane 1, protein purified with Ni-NTA column from the soluble fraction in the bacterial lysate; lane 2, the molecular weight markers. Gel was stained with silver staining.
(1.50 MB TIF)

Figure S2 DNA-sequence similarity comparison of l-paf-ah from L. interrogans serovar Lai strain 56601 (la2144) with that from the avirulent strain of L. interrogans serovar Lai (Avirulent Lai), L. interrogans serovar Javanica strain M 10 (Javanica), L. biflexa serovar Montevalerio (Montevalerio), L. interrogans serovar Linhai strain Lin 6 (Linhai), L. interrogans serovar Paidjan strain L 37 (Paidjan), L. interrogans serovar Pomona strain Luo (Pomona), L. interrogans serovar Hebdomadis strain P 7 (Hebdomadis), L. biflexa serovar Anhui strain Zong 7 (Anhui), L. interrogans serovar Canicola strain Lin (Canicola).
(8.36 MB TIF)

Figure S3 Study design in gerbil leptospirosis model.
(3.53 MB TIF)

Figure S4 PCR assay detection of leptospira on the 4th day in lung, heart, kidney, spleen and liver of gerbils inoculated with PBS, the avirulent strain of L. interrogans serovar Lai and L. interrogans serovar Lai. Lane 1, injected with PBS alone; lane 2, injected with the avirulent strain of L. interrogans serovar Lai; lane 3, injected with L. interrogans serovar Lai; lane M, DNA molecular size marker.
(0.92 MB TIF)

Figure S5 Time-course of L-PAF-AH in the sera of gerbils injected with the virulent strain. The measurements were performed on the 2nd, 3rd, 4th, 5th, 6th, 7th, 8th and 9th days via both ELISA and immunoprecipation followed by mass spectrometric identification. The EMJH culture supernatant of leptospira obtained in the later stationary phase was used as the positive controls. ⧫, L-PAF-AH detected by ELISA; ◊, L-PAF-AH undetectable by ELISA; ★, L-PAF-AH detected by immunoprecipation followed by mass spectrometry; ⋆, L-PAF-AH undetectable by immunoprecipation followed by mass spectrometry. The standard error of the experiments was indicated by bars.
(0.19 MB TIF)

Figure S6 PAF-AH levels in serum from the 1st to 9th day in gerbils with experimentally infected leptospirosis and in healthy gerbils. PBS (n = 107), injected with PBS alone; avirulent (n = 106), injected with the avirulent strain of L. interrogans serovar Lai; virulent (Total) (n = 136), injected with L. interrogans serovar Lai, including the serum of the gerbils which were dying and 5 hr before dying; virulent (Dying) (n = 56), injected with PBS with L. interrogans serovar Lai, and collected from the gerbils which were dying and 1 hr before dying. Horizontal bars represented the mean value for each group.
(0.23 MB TIF)
