# Supplementary figures and images for: Serum Activity of Platelet-Activating Factor Acetylhydrolase Is a Potential Clinical Marker for Leptospirosis Pulmonary Hemorrhage
Source: PLoS One. 2009 Jan 15;4(1):e4181. doi: 10.1371/journal.pone.0004181 (PMC2615213; doi:10.1371/journal.pone.0004181)

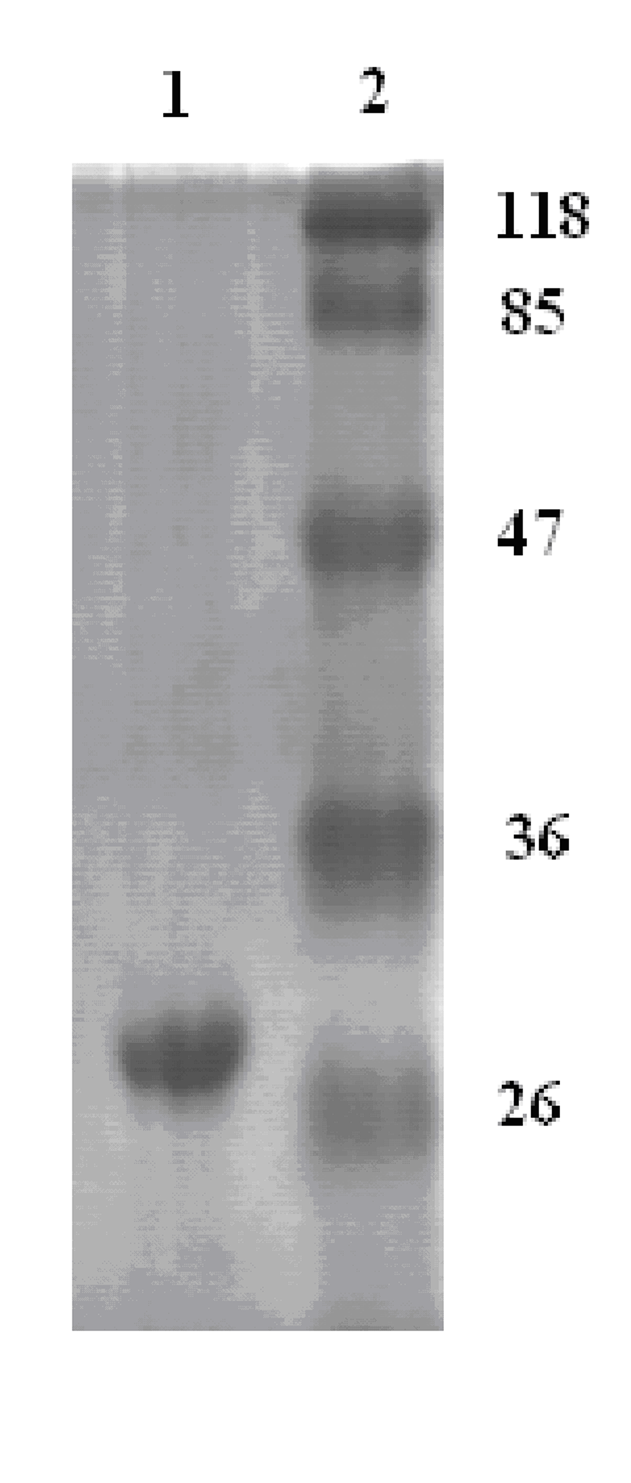

Supplement: Figure S1 — Purification of His-Tag fusion protein L-PAF-AH in the E. coli BL21 (DE3) pLysS cells monitored with the 15% SDS-PAGE. Lane 1, protein purified with Ni-NTA column from the soluble fraction in the bacterial lysate; lane 2, the molecular weight markers. Gel was stained with silver staining. (1.50 MB TIF) [file pone.0004181.s001.tif]

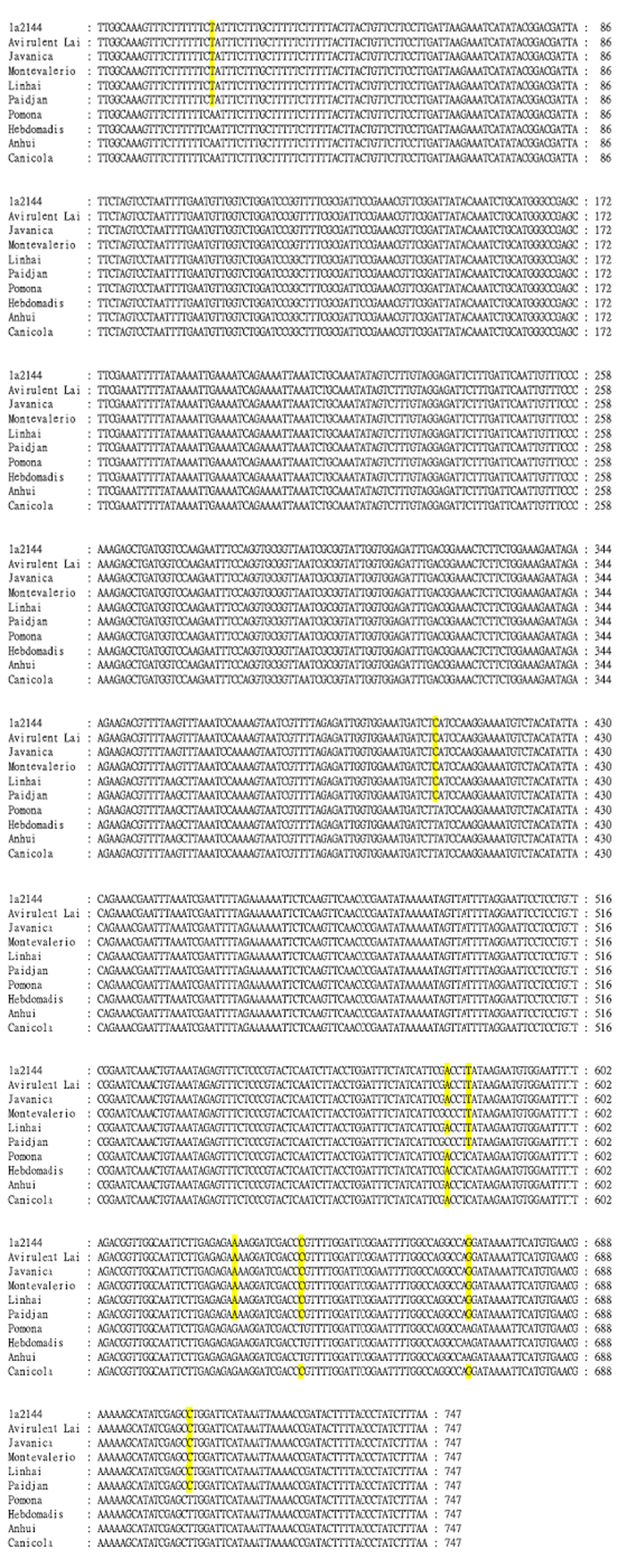

Supplement: Figure S2 — DNA-sequence similarity comparison of l-paf-ah from L. interrogans serovar Lai strain 56601 (la2144) with that from the avirulent strain of L. interrogans serovar Lai (Avirulent Lai), L. interrogans serovar Javanica strain M 10 (Javanica), L. biflexa serovar Montevalerio (Montevalerio), L. interrogans serovar Linhai strain Lin 6 (Linhai), L. interrogans serovar Paidjan strain L 37 (Paidjan), L. interrogans serovar Pomona strain Luo (Pomona), L. interrogans serovar Hebdomadis strain P 7 (Hebdomadis), L. biflexa serovar Anhui strain Zong 7 (Anhui), L. interrogans serovar Canicola strain Lin (Canicola). (8.36 MB TIF) [file pone.0004181.s002.tif]

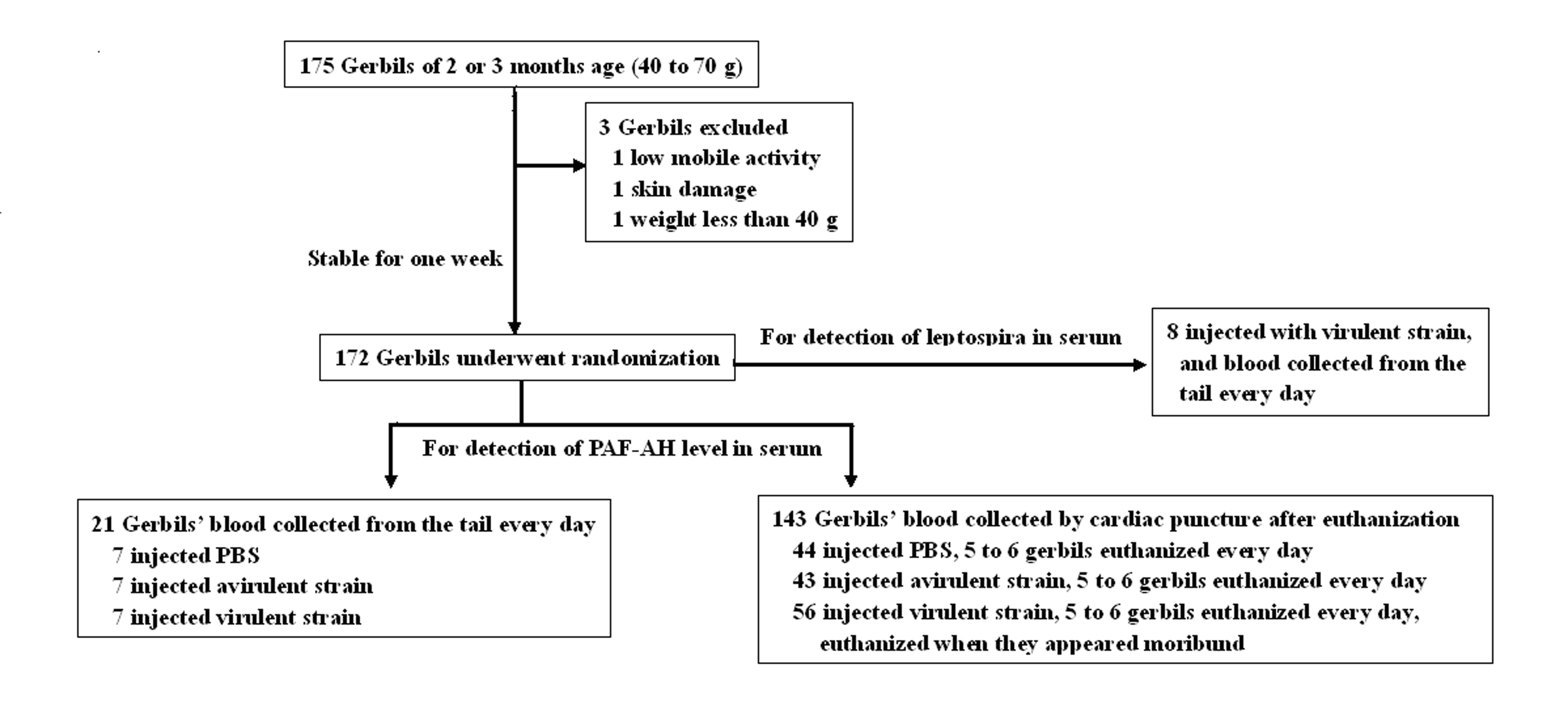

Supplement: Figure S3 — Study design in gerbil leptospirosis model. (3.53 MB TIF) [file pone.0004181.s003.tif]

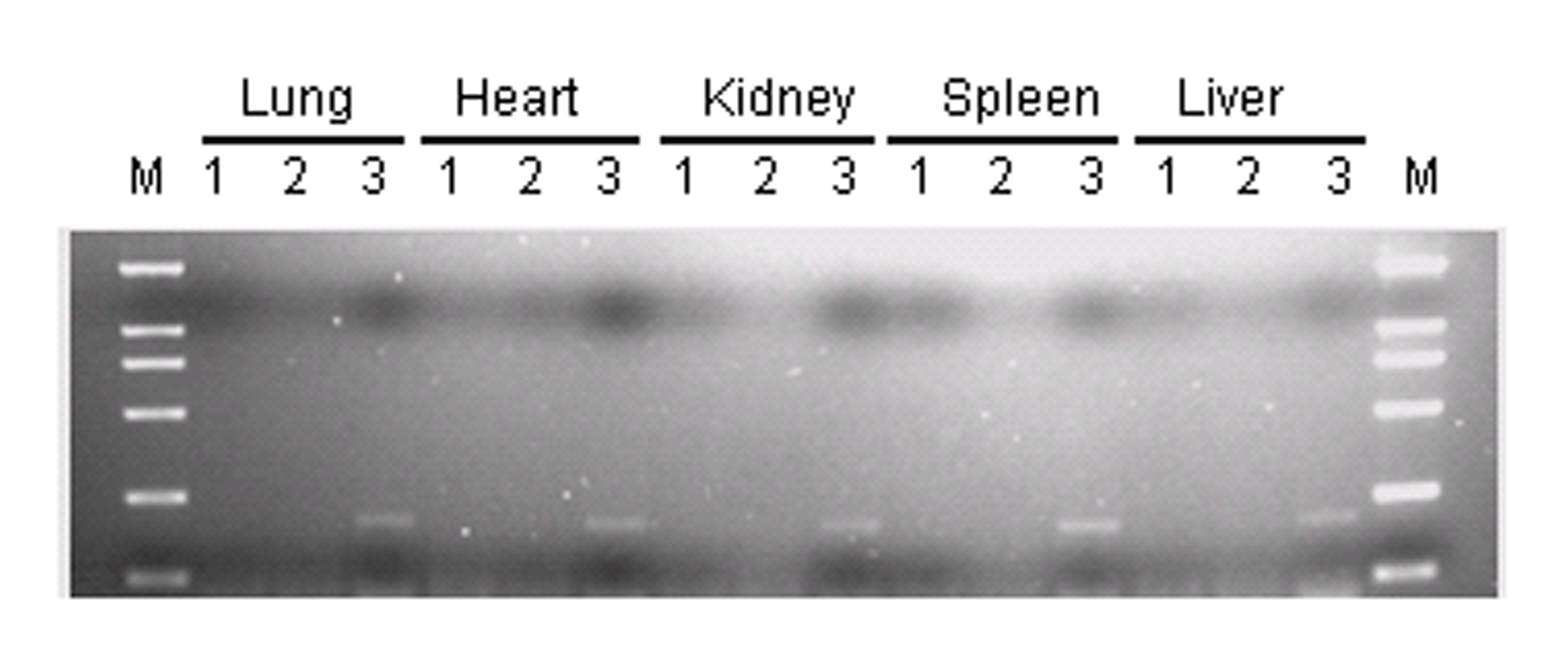

Supplement: Figure S4 — PCR assay detection of leptospira on the 4th day in lung, heart, kidney, spleen and liver of gerbils inoculated with PBS, the avirulent strain of L. interrogans serovar Lai and L. interrogans serovar Lai. Lane 1, injected with PBS alone; lane 2, injected with the avirulent strain of L. interrogans serovar Lai; lane 3, injected with L. interrogans serovar Lai; lane M, DNA molecular size marker. (0.92 MB TIF) [file pone.0004181.s004.tif]

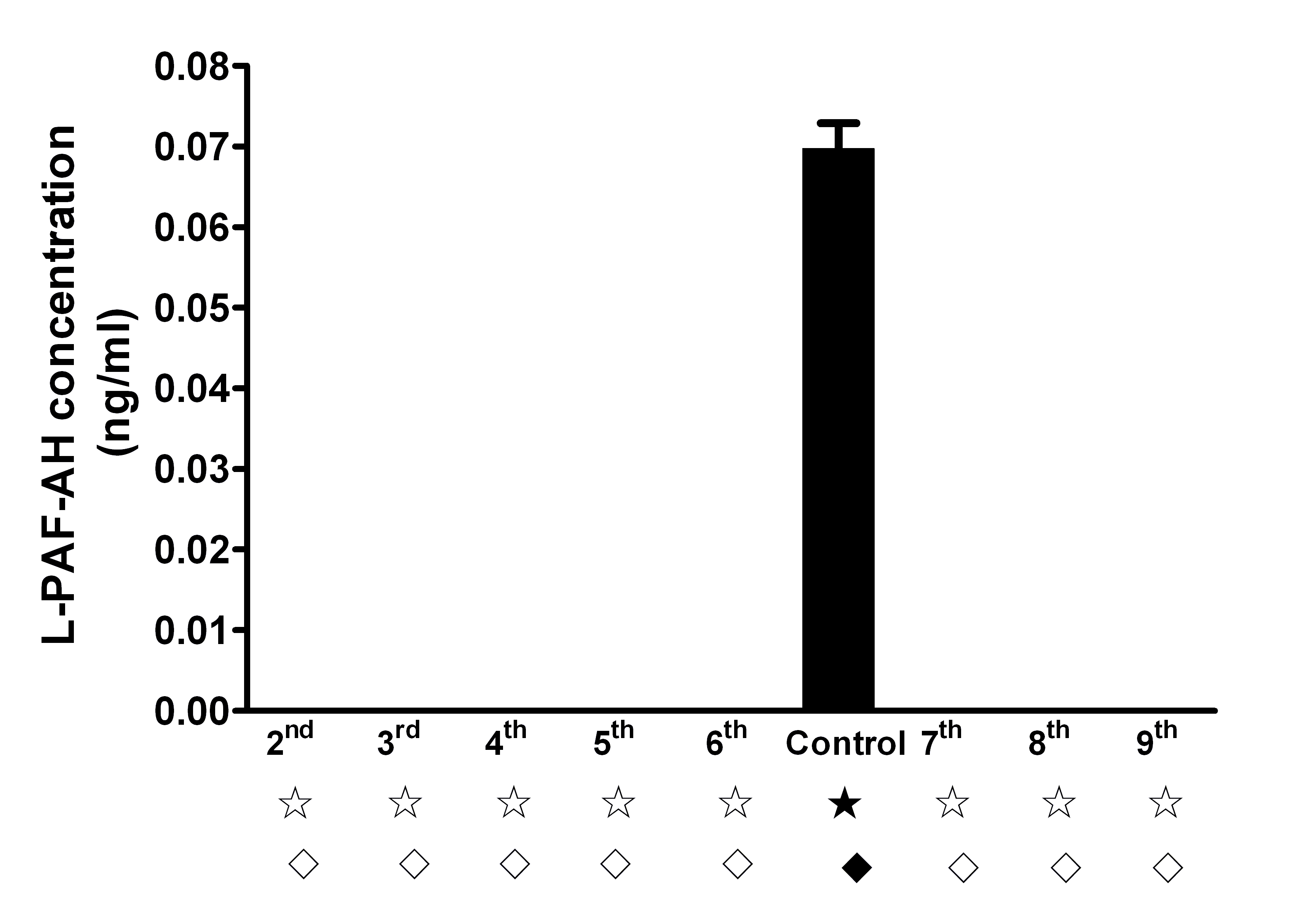

Supplement: Figure S5 — Time-course of L-PAF-AH in the sera of gerbils injected with the virulent strain. The measurements were performed on the 2nd, 3rd, 4th, 5th, 6th, 7th, 8th and 9th days via both ELISA and immunoprecipation followed by mass spectrometric identification. The EMJH culture supernatant of leptospira obtained in the later stationary phase was used as the positive controls. ⧫, L-PAF-AH detected by ELISA; ◊, L-PAF-AH undetectable by ELISA; ★, L-PAF-AH detected by immunoprecipation followed by mass spectrometry; ⋆, L-PAF-AH undetectable by immunoprecipation followed by mass spectrometry. The standard error of the experiments was indicated by bars. (0.19 MB TIF) [file pone.0004181.s005.tif]

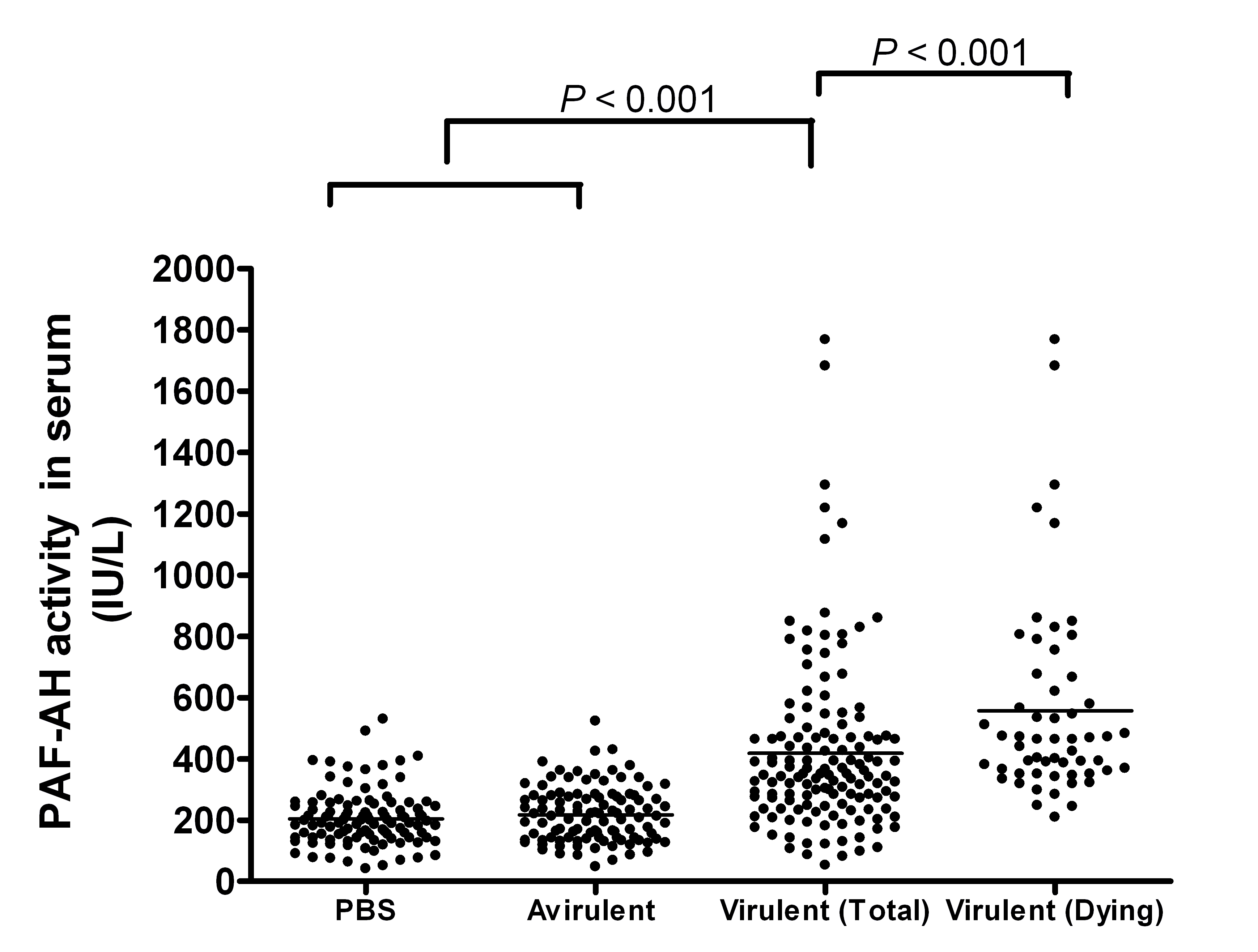

Supplement: Figure S6 — PAF-AH levels in serum from the 1st to 9th day in gerbils with experimentally infected leptospirosis and in healthy gerbils. PBS (n = 107), injected with PBS alone; avirulent (n = 106), injected with the avirulent strain of L. interrogans serovar Lai; virulent (Total) (n = 136), injected with L. interrogans serovar Lai, including the serum of the gerbils which were dying and 5 hr before dying; virulent (Dying) (n = 56), injected with PBS with L. interrogans serovar Lai, and collected from the gerbils which were dying and 1 hr before dying. Horizontal bars represented the mean value for each group. (0.23 MB TIF) [file pone.0004181.s006.tif]
